# Minichromosome maintenance 4 is associated with poor survival and stemness of patients with pancreatic cancer

**DOI:** 10.1007/s00795-025-00438-y

**Published:** 2025-04-28

**Authors:** Yuto Fujiki, Akira Ishikawa, Narutaka Katsuya, Yuki Shiwa, Takafumi Fukui, Kazuya Kuraoka, Takeshi Sudo, Sho Tazuma, Yasutaka Ishii, Shiro Oka, Wataru Yasui, Shinji Mii

**Affiliations:** 1 Department of Molecular Pathology, Graduate School of Biomedical and Health Sciences, Hiroshima University, 1-2-3 Kasumi, Minami-ku, Hiroshima, 734-8551 Japan; 2 Department of Diagnostic Pathology, National Hospital Organization (NHO), Kure Medical Center, and Chugoku Cancer Center, 3-1 Aoyama, Kure, 737-0023 Japan; 3 Department of Surgery, National Hospital Organization (NHO), Kure Medical Center, and Chugoku Cancer Center, 3-1 Aoyama, Kure, 737-0023 Japan; 4 Department of Gastroenterology, Graduate School of Biomedical and Health Sciences, Hiroshima University, 1-2-3 Kasumi, Minami-ku, Hiroshima, 734-8551 Japan; 5 Division of Pathology, Hiroshima City Medical Association Clinical Laboratory, 3 Chome-8-6 Sendamachi, Naka-ku, Hiroshima, 730-8611 Japan

**Keywords:** Pancreatic cancer, MCM4, Cancer stem cell, Molecular pathology, Single cell analysis

## Abstract

Pancreatic ductal adenocarcinoma (PDAC) is one of the most well-known cancer types, with a persistently poor 5-year survival rate. We previously reported MCM4 as a molecule associated with cancer stem cells; however, its role in PDAC has not been reported. Therefore, in this study, we aimed to fill this gap in the literature. We analyzed MCM4 expression in 81 PDAC samples using immunohistochemistry (IHC). The functional role of MCM4 in PDAC was investigated using RNA interference in PDAC cell lines. Additionally, a single-cell analysis was conducted by downloading data from six PDAC cases. On IHC, high MCM4 expression was observed in 42 out of 81 (51.9%) PDAC cases. MCM4-positive PDAC was significantly associated with a higher pN grade. Furthermore, high MCM4 expression was linked to a significantly poorer prognosis and was identified as an independent prognostic factor in multivariate analysis. In PDAC cell lines, MCM4 knockdown impairs cell growth and spheroid formation. Single-cell analysis also revealed that MCM4-expressing cells were located upstream of the trajectory, with a cluster showing a correlation with KIFC1, which has been reported to be associated with cancer stemness. These results indicated the significance of MCM4 expression in PDAC and its association with cancer stemness.

## Introduction

Pancreatic ductal adenocarcinoma (PDAC) is the most common pancreatic malignancy, with a dismal 5-year survival rate of just around 11%, despite recent advancements in understanding PDAC tumor biology and the development of novel therapies [1]. Although PDAC can take time to progress [2], making early detection relatively manageable for treatment [3], only approximately 10–20% of patients have resectable pancreatic cancer at diagnosis [1]. Chemotherapy remains the cornerstone of PDAC treatment. Although some patients experience substantial clinical benefits, therapeutic resistance prevails. This resistance highlights the resilience of pancreatic cancer and its remarkable ability to create a tumor-protective microenvironment [4]. Therefore, in PDAC treatment strategies, the development of additional molecular targeted therapies is highly desirable, and targeting a distinctive microenvironment is considered a promising approach.

Targeting cancer stem cells (CSCs), a key component of the tumor microenvironment, offers a means of understanding their pathogenesis and identifying potential therapeutic targets. CSCs are believed to drive tumor initiation and sustain self-renewal and are closely associated with chemotherapy resistance, recurrence, and metastasis [5]. CSC markers, such as CD133, CD24, CD44, DCLK1, and CXCR4, have been identified in PDAC [6] and are correlated with the clinical stage, among other factors. Cells expressing these CSC markers exhibit high tumorigenicity [7] and may serve as potential therapeutic targets. However, many aspects of the molecular mechanisms and characteristics of CSCs in PDAC remain unclear. Previously, through spheroid analysis, we demonstrated that KIFC1 [8] and IQGAP3 [9] are associated with cancer stem cell properties in gastric cancer and reported that these molecules may also serve as CSC markers in PDAC [10, 11]. Our subsequent focus was on the molecule minichromosome maintenance 4 (MCM4).

MCM4 belongs to the minichromosome maintenance (MCM) protein family and is only observed during chromosomal replication [12, 13]. The MCM complex is a crucial macromolecular machine required for DNA replication in eukaryotes and archaea [14]. MCM4 is an essential component of the MCM2–7 complex, indispensable for initiating genome replication in eukaryotic cells [15]. Within this hexameric complex, MCM4 exhibits ATPase activity and plays a central role in unwinding the DNA double helix during replication [16]. Moreover, it is crucial for replication fork formation and progression to work with other MCM subunits to ensure accurate DNA synthesis during the S phase. Together with its partner proteins, MCM4 operates as part of the core replicative helicase, driving the separation of parental DNA strands and enabling bidirectional replication [17]. MCM4 is also expressed in various carcinomas [15], with reports indicating that high expression is associated with poor prognosis in the breast [18], liver [19], and urothelial carcinomas [20]. We focused on MCM4 as a molecule associated with CSC properties in gastric cancer and demonstrated its association with poor prognosis through clinical specimens and cellular experiments [21]. Only one study has reported that high MCM4 expression is a marker of poor prognosis based on early PDAC mRNA levels [22]. However, no research has examined this marker using immunohistochemistry or from the perspective of CSC characteristics.

Therefore, we investigated the clinical significance of MCM4 in PDAC, analyzed its correlation with other molecules associated with tumor progression, and confirmed the biological function of MCM4 using MCM4 knockdown PDAC cell lines. In addition, we reanalyzed single-cell data from public databases. These findings offer new insights into the use of MCM4 as a potential biomarker of PDAC.

## Materials and methods

### Tissue samples

A consecutive cohort of 81 histopathologically confirmed PDAC patients who underwent surgical resection at the Kure Medical Center and Chugoku Cancer Center (Hiroshima, Japan) between April 1, 2015, and March 31, 2020, was included in the study, as previously described [11]. Archived formalin-fixed, paraffin-embedded tumor tissues from resected specimens were used for immunohistochemical analyses. One representative tumor block from each specimen was assessed via IHC, and histological classifications were determined according to the World Health Organization classification [23]. Tumor assessment was performed according to the criteria defined in the Union for International Cancer Control tumor-node-metastasis classification guide [24]. Informed consent was obtained from all patients. 

### Immunohistochemistry (IHC)

Immunohistochemical analyses were performed using a Dako Envision + Mouse Peroxidase Detection System (Dako Cytomation, Carpinteria, CA, USA). The details of MCM4 immunohistochemical staining have been described in a previous publication [21]. Sections were incubated with a mouse polyclonal anti-MCM4 antibody (dilution, 1:200) for 1 h at room temperature, followed by incubation with Envision + anti-mouse peroxidase for 1 h. The expression of MCM4 in PDAC was scored as positive or negative for all tumors. When more than 5% of tumor cells were stained, immunostaining was considered positive for MCM4 as our previous publication [21]. Using these definitions, two observers (F. Y. and I. A.), who did not know the clinical and pathologic parameters or patient outcomes, independently reviewed the immunoreactivity of each specimen. The expression of ANXA10 (Annexin A10) [25], IQGAP3, CD44, CD133, Ki-67 [11], CLDN18, PDX1, and KIFC1 [10] was scored in all tumors as positive or negative, as previously described. All antibody vendors and dilutions are summarized in Table 1.

**Table 1:** Primary antibodies for immunohistochemistry (IHC) and western blot (WB)

Antibody	Catalog no	Vendor	Dilution (IHC)	Dilution (WB)
MCM4	SC-28317	Santa Cruz	1:200	1:1000
Ki-67	M724029-2	Dako	Pre-diluted	
ANXA10	NBP1-90,156	Novus Biologicals	1:500	
IQGAP3	ab219354	Abcam	1:100	
CD44	ab270255	Abcam	1:100	
CD133	130–090–422	Miltenyi Biotec	1:100	
CLDN18	38–8000	Invitrogen	1:200	
PDX1	ab134150	Abcam	1:1000	
KIFC1	H00003833-M01	Abnova	1:100	
Beta actin	A5441	Sigma		1:1000

### Cell lines

The human PDAC cell lines PANC-1, PK-1, KP-4, and T3M-4 were purchased from the Japanese Collection of Research Bioresources Cell Bank (Osaka, Japan). All cell lines were maintained at 37 °C in Dulbecco’s modified Eagle’s medium (DMEM) medium (Thermo Fisher Scientific, Waltham, MA, USA) containing 10% fetal bovine serum (Corning, Corning, NY, USA) in a humidified atmosphere with 5% CO_2_.

### Western blotting (WB)

In western blotting analysis, lysates (30 µg) were solubilized in Laemmli buffer by boiling and then subjected to 10% sodium dodecyl sulfate–polyacrylamide gel electrophoresis followed by electrotransfer onto a nitrocellulose filter. The membrane was then incubated with a primary antibody against MCM4 (1:1000 dilution). Peroxidase-conjugated anti-mouse IgG was used for the secondary reaction. The immunocomplexes were visualized using an ECL western blot detection system (Amersham Biosciences). β-Actin (Sigma, St. Louis, MO, USA) was used as a loading control.

### RNA interference

Small interfering RNA (siRNA) oligonucleotides targeting MCM4 and negative control siRNA were obtained from Invitrogen (Carlsbad, CA, USA). Transfection was performed using Lipofectamine RNAiMAX (Invitrogen) as previously described [21]. In brief, 60 pmol of siRNA and 10 μL of Lipofectamine RNAiMAX were combined in 1 mL of RPMI medium. After a 20-min incubation, the mixture was added to the cells, which were then seeded in culture dishes. All experiments were performed 48 h post-transfection.

### Cell growth assays

Cell growth was examined using 3-(4,5-dimethylthiazol-2-yl)−2,5-diphenyltetrazolium bromide assay. Cells were seeded at a density of 4000 cells/well in 96-well plates. Cell growth was monitored after 1, 2, and 4 days. Three independent experiments were performed. Mean and standard deviation (S.D.) were calculated for each experiment.

### Spheroid colony formation assay

For spheroid generation, the PDAC cell line T3M-4 was used; 200 cells were seeded in 6-well ultra-low attachment plates (Corning). Cells were grown in mTeSR medium (STEMCELL Technologies Inc., Vancouver, BC, Canada). The plates were incubated at 37 °C in an incubator with a 5% CO_2_ atmosphere for 7 days. Spheroid number and size were determined using a microscope, as previously described [21].

### Quantitative reverse transcription-polymerase chain reaction (qRT-PCR)

Total RNA was extracted using an RNeasy Mini kit (Qiagen, Valencia, CA, USA), and 1 μg of total RNA was converted to cDNA using a First-Strand cDNA Synthesis kit (Amersham Biosciences, Piscataway, NJ, USA). Quantitative RT-PCR was performed using a CFX Opus 96 Real-Time PCR Instrument (#12011319 J1) at the Natural Science Center for Basic Research and Development, Hiroshima University. We examined the expression of *MCM4* and the control gene *ACTB* by qRT-PCR amplification using the SYBR Select Master Mix (Applied Biosystems, Foster City, CA). The *MCM4* and *ACTB* primer sequences used for qRT-PCR were as follows: *MCM4* -F AATGTACTGCTGCTTGGCCT, R GGCTGGACCGAGAGAGTTTC; *ACTB* -F CGGGAGAAATTGCAGGAGGA, R AAGGTCAAGACGTGCCAGAG; *CLDN18* -F TGGAGTGTCTGTGTTTGCCA, R GGTCTGAACAGTCTGCACCA; *PDX1* -F GACATCTCCCCGTACGAGGT, R GGAGGTGGTGGTGAAGGTG; *KIFC1* -F GACGCCCTGCTTCATCTG, -R CCAGGTCCACAAGACTGAGG.

### Single-cell RNA sequencing data sets and analysis

We used single-cell datasets from the public dataset GSE212966 [26], which contained six PDAC cases. The gene-cell matrices were imported into R software (v4.4.2) to further filter out low-quality cells (< 100 genes, > 20% mitochondrial genes, > 50,000 transcripts, and > 5000 genes) using the “Seurat” R package (v5.1.0), which are generally consistent with those described in our previous report [27]. In total, 31,263 cells were extracted from six PDAC cases, revealing 26,735 features. Furthermore, EPCAM expression was used as a marker to isolate PDAC cells, extracting 4,260 cells that were identified as PDAC cells. Gene expression levels were normalized (LogNormalize) with “NormalizedData.” A total of 2000 highly variable genes were selected and used for PCA reduction. Moreover, t-distributed stochastic neighbor embedding (t-SNE) was performed. Dimensionality reduction and clustering were performed using independent component analysis, uniform manifold approximation, and projection (UMAP). Computational analysis of compartment embedding trajectories was performed using the"Monocle 3"(version 1.3.7) algorithm. Gene Set Enrichment Analysis (GSEA) was performed using the"Molecular Signatures Database (MSigDB)"package (version 7.5.1).

### Statistical methods

Correlations between clinicopathological features and *MCM4* expression were analyzed using Fisher’s exact test. Kaplan–Meier survival curves were constructed for *MCM4*-positive and -negative cases and survival rates were compared between the positive and negative groups. Differences between the survival curves were tested for statistical significance using the log-rank test. Univariate and multivariate Cox proportional hazard regression analyses were used to evaluate the association between clinical covariates and survival. Differences between the two groups (*MCM4* siRNA-transfected cells and negative control siRNA-transfected cells) in each assay were tested using an unpaired *t* test. Age (cutoff: 74 years) and tumor size (cutoff: 2.8 cm) were analyzed using median-based stratification. Statistical significance was set at *p* < 0.05. significance.

## Results

### Expression and prognostic significance of MCM4 in PDAC

**Fig. 1:**
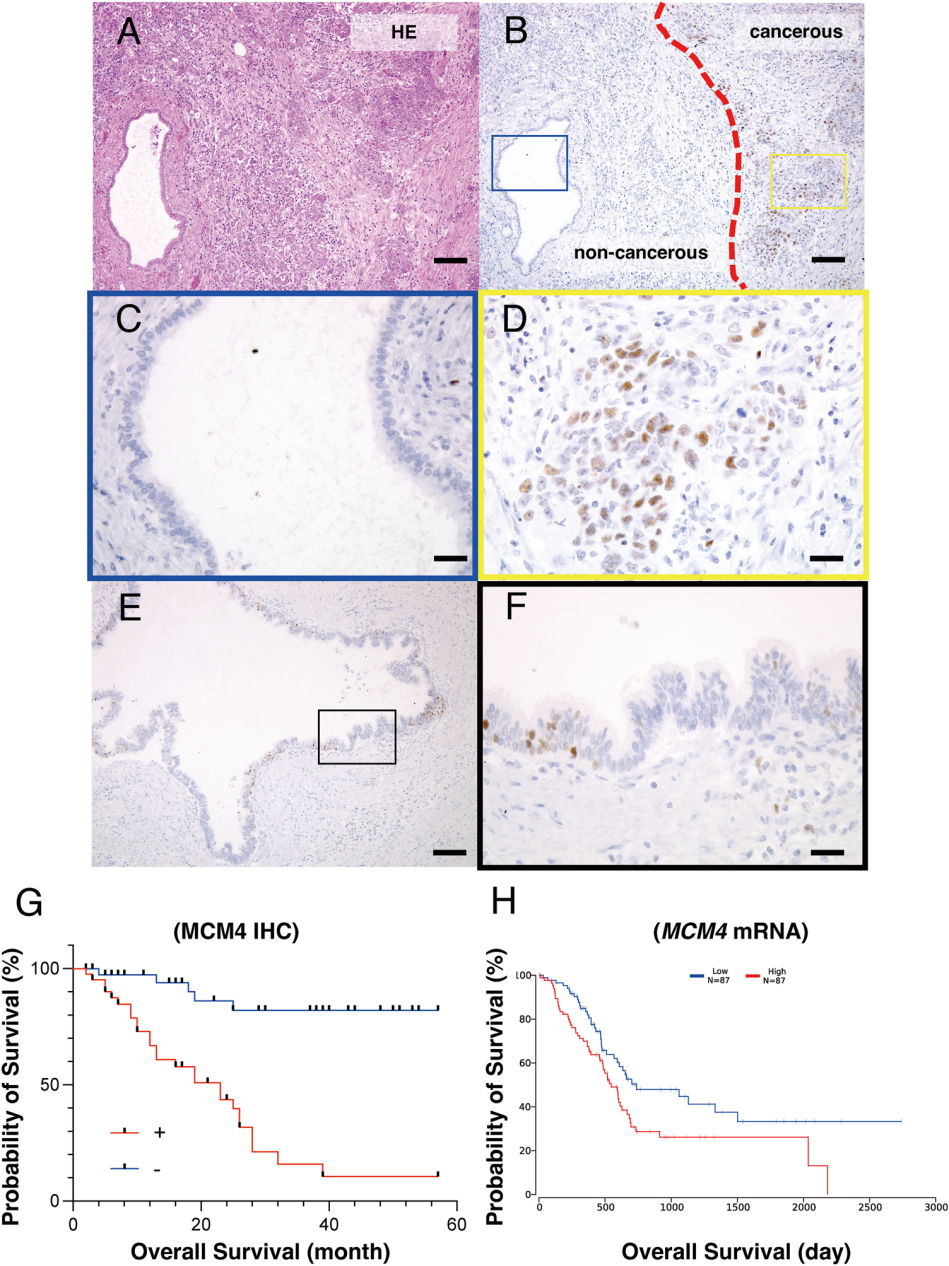
Expression of MCM4 in pancreatic ductal cancer (PDAC). **A** Representative corresponding HE image. **B–D** Representative immunohistochemical images of MCM4 in PDAC cases. Original magnification, **B** × 40; scale bars, 500 μm and **C**, **D** × 400; scale bars, 50 μm. **E**, **F** Representative immunohistochemical images of MCM4 of high-grade pancreatic intraepithelial neoplasia. Original magnification, **E** × 100; scale bars, 200 μm and **F** × 400; scale bars, 50 μm. **G** Kaplan–Meier plot of overall survival based on MCM4 expression. **H** Kaplan–Meier plot of overall survival based on The Cancer Genome Atlas data

We performed immunohistochemical staining to examine MCM4 localization in 81 surgically resected PDAC tissue samples. MCM4 expression was distinctly observed in cancerous and non-cancerous regions (Fig. 1A, B). MCM4 was not expressed in non-cancerous pancreatic ductal cells (Fig. 1C). MCM4 expression was observed in the nuclei of PDAC cells and was detected in 42 (51.9%) out of 81 cases (Fig. 1D). Additionally, MCM4 expression was observed in the nuclei of high-grade pancreatic intraepithelial neoplasia (pancreatic carcinoma in situ; Fig. 1E, F); however, its distribution was heterogeneous, with areas of clustered expression and areas lacking expression. This randomness was also observed in invasive carcinoma. Next, we investigated the correlation between MCM4 staining and the clinicopathological characteristics of 81 PDAC cases (Table 2). MCM4-positive cases were significantly associated with the pN stage (*p* = 0.012) and pStage (*p* < 0.01). No significant differences were observed in other characteristics (age, sex, tumor size, location, histology, venous and lymphatic invasion, and perineural invasion) between MCM4-negative and MCM4-positive cases.

**Table 2:** The relationship between MCM4 expression and clinicopathological features in patients with pancreatic invasive ductal carcinoma (PDAC)

	MCM4 expression		*p* value
	Positive (%)	Negative	
Age (years)			0.377
< 75	24 (57)	18	
≥ 75	18 (46)	21	
Sex			0.269
Male	25 (60)	18	
Female	17 (45)	21	
Tumor size (cm)			0.268
< 2.8	19 (45)	23	
≥ 2.8	23 (59)	16	
Location			0.659
Ph	20 (49)	21	
Pb/Pt	22 (55)	18	
Grade (G)			0.076
G1	14 (40)	21	
G2/G3	28 (61)	18	
pStage			
IIA or lower	8 (30)	19	< 0.01
IIB or higher	34 (63)	20	
pN			0.012
pN0	9 (32)	19	
pN1/2	33 (62)	20	
Venous invasion (V)			0.2664
V0	15 (44)	19	
V1	27 (57)	20	
Lymphatic invasion (L)			0.320
L0	29 (48)	31	
L1	13 (62)	8	
Perineural invasion (Pn)			0.8217
Pn0	27 (53)	24	
Pn1	15 (50)	15	
Resection margin (R)			0.370
R0	37 (54)	31	
R1	5 (38)	8	

In the analysis of the 5-year overall survival, MCM4-positive PDAC cases demonstrated significantly poorer survival probabilities than did MCM4-negative cases (*p* < 0.01; Fig. 1D). These results were further supported by TCGA data available from public databases (*p* < 0.01; Fig. 1E). Univariate and multivariate Cox proportional hazard analyses were performed to further assess MCM4 as a prognostic classifier (Table 3). In univariate analysis, N grade (HR, 2.635; 95% CI 1.076–6.453; *p* = 0.0340), pStage (HR, 2.630; 95% CI 1.074–6.443; *p* = 0.0343), and MCM4 expression (HR, 8.270; 95% CI 3.126–27.87; *p* < 0.01) were associated with poor survival. As for multivariate analysis, only MCM4 expression (HR, 7.418; 95% CI 2.744–20.05; *p* < 0.01) was shown to be an independent predictor in PDAC cases. These findings suggest that MCM4 plays a role in PDAC progression and is an independent prognostic factor following surgical resection.

**Table 3:** Univariate and multivariate Cox proportional hazard regression analyses of MCM4 expression and survival of pancreatic cancer patients

Features	Univariate analysis		Multivariate analysis	
	HR (95% CI)	*p* value	HR (95% CI)	*p* value
Age		0.114		
≤ 74	1 (ref.)			
> 74	1.772 (0.872–3.601)			
Location		0.646		
Ph	1 (ref.)			
Pb/Pt	1.180 (0.582–2.394)			
Grade (G)		0.0518		
G1	1 (ref.)			
G2/G3/G4	2.115 (0.994–4.500)			
pN		0.0340		0.328
pN0	1 (ref.)		1 (ref.)	
pN1/2	2.635 (1.076–6.453)		1.584 (0.630–4.000)	
pStage		0.0343		
IIA or lower	1 (ref.)			
IIB or higher	2.630 (1.074–6.443)			
Lymphatic invasion (L)		0.482		
L0	1 (ref.)			
L1	1.322 (0.607–2.879)			
Venous invasion (V)		0.150		
V0	1 (ref.)			
V1	1.716 (0.823–3.576)			
Size		0.160		
< 2.8 cm	1 (ref.)			
≥ 2.8 cm	1.672 (0.816–3.426)			
Resection margin (R)		0.201		
	1 (ref.)			
	2.174 (0.660–7.160)			
MCM4 expression		< 0.01		< 0.01
Negative	1 (ref.)		1 (ref.)	
Positive	8.270 (3.126–27.87)		7.418 (2.744–20.05)	

### Correlation between MCM4 expression and other molecules

**Fig. 2:**
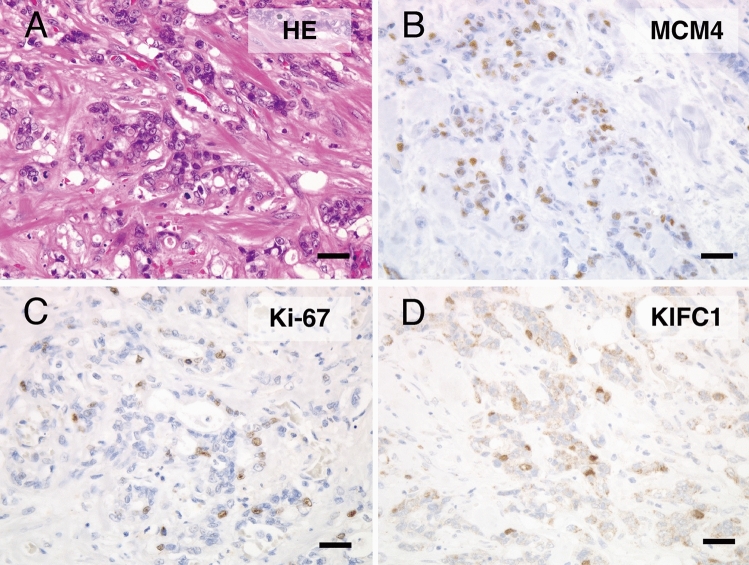
Representative immunohistochemical images of HE, MCM4, Ki-67, and KIFC1. **A** HE, **B** MCM4, **C** Ki-67, and **D** KIFC1. Original magnification: 400 ×; scale bars, 50 μm

Our data suggest that MCM4 plays a crucial role in PDAC progression. To evaluate the association between MCM4 and other molecules, we conducted immunohistochemical analyses for Ki-67, ANXA10, IQGAP3, CLDN18, PDX1, and cancer stem cell markers CD44 and CD133 using the same cohort of PDAC cases that had been stained for MCM4 (Table 4). Representative hematoxylin and eosin (HE; Fig. 2A) demonstrated that the expression of MCM4 (Fig. 2B) was significantly associated with those of Ki-67 (*p* < 0.01; Fig. 2C) and KIFC1 (*p* < 0.01; Fig. 2D), the latter of which has been previously identified as a cancer stem-like molecule [10]. In contrast, MCM4 expression was inversely correlated with CLDN18 (*p* < 0.01) and PDX1 (*p* = 0.045). No significant associations were observed between ANXA10, IQGAP3, CD44, or CD133. These results suggested that MCM4 expression is linked with cell proliferation and certain CSC-like properties.

**Table 4:** The relationship between MCM4 expression and several molecules including cancer stem cell or cell proliferation markers in patients with pancreatic cancer

	MCM4 expression		
	Positive (%)	Negative	*p* value
Ki-67			< 0.01
Positive	34 (71)	14	
Negative	8 (24)	25	
ANXA10			0.266
Positive	27 (57)	20	
Negative	15 (44)	19	
IQGAP3			0.508
Positive	24 (56)	19	
Negative	18 (47)	20	
CD44			0.931
Positive	22 (59)	15	
Negative	20 (41)	24	
CD133			0.592
Positive	10 (59)	7	
Negative	32 (50)	32	
CLDN18			< 0.01
Positive	15 (34)	29	
Negative	27 (72)	10	
PDX1			0.0457
Positive	14 (39)	22	
Negative	28 (62)	17	
KIFC1			< 0.01
Positive	26 (70)	11	
Negative	16 (36)	28

### Effect of MCM4 inhibition on PDAC cells

**Fig. 3:**
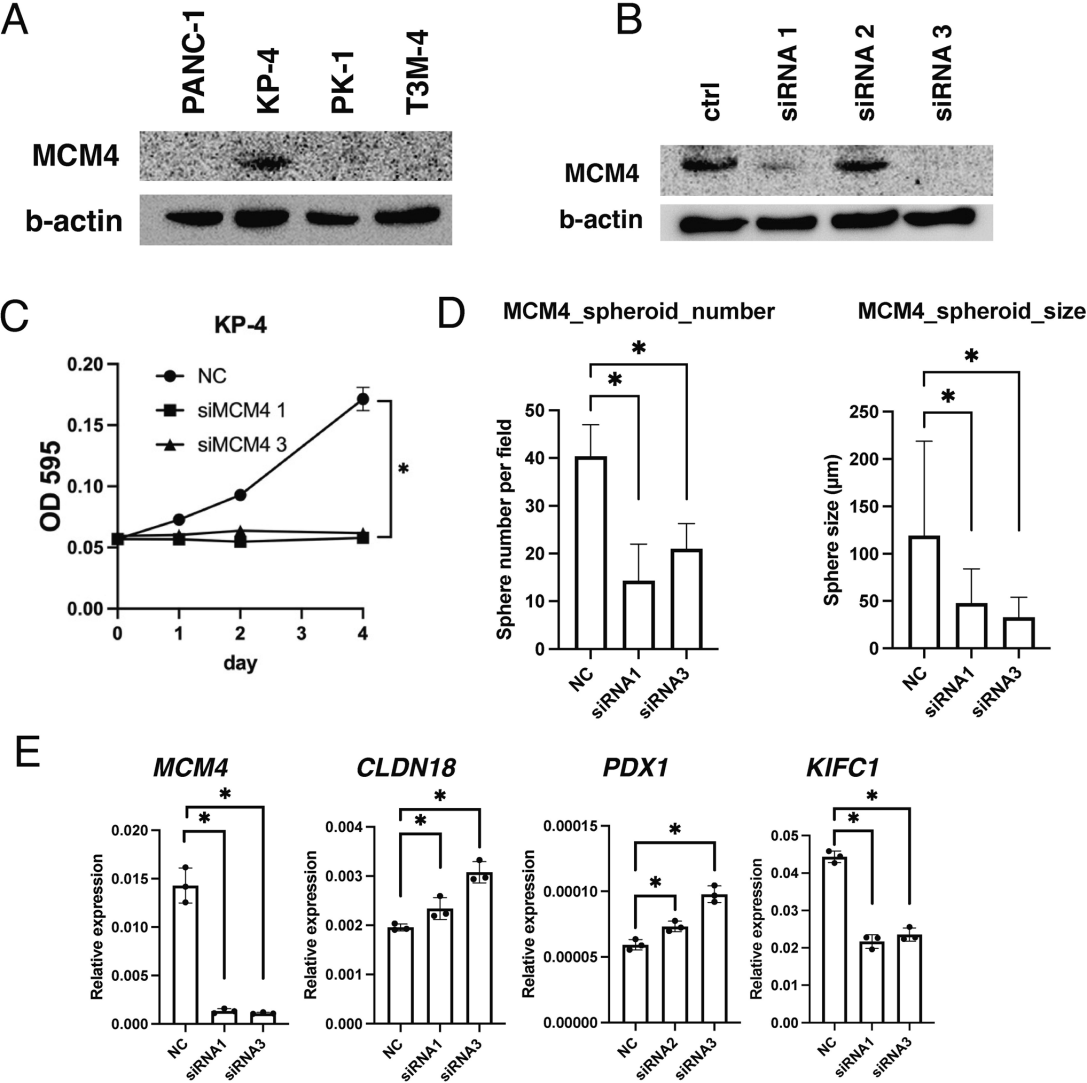
Effect of MCM4 inhibition in PDAC cells. **A** Western blot analysis of MCM4 in 4 PDAC cell lines. **B** Western blot analysis of KIFC1 in KP-4 cells transfected with the negative control or MCM4 siRNA. **C** Effect of KIFC1 knockdown on cell growth in PDAC cells transfected with the negative control or MCM4 siRNA. **D** Number and size of spheroids formed by KP-4 cell lines transfected with negative control or MCM4 siRNA. **E** Quantitative reverse transcription-polymerase chain reaction analysis of MCM4, CLDN18, PDX1, and KIFC1 genes in KP-4 cells transfected with the negative control or MCM4 siRNA

To validate these findings, we conducted functional analyses of the PDAC cell lines. High MCM4 expression was observed in KP-4 cells (Fig. 3A). We confirmed the knockdown of MCM4 in KP-4 cells using MCM4-specific siRNA by WB (Fig. 3B). Subsequently, a proliferation assay was performed using these MCM4 knockdown cell lines. The growth of MCM4 knockdown cells was significantly reduced compared to that of the negative control siRNA-transfected cells (Fig. 3C). In addition, we examined the association between MCM4 expression and spheroid colony formation in PDAC cell lines. Both the number and size of spheroids significantly decreased in MCM4 siRNA-transfected KP-4 cells (Fig. 3D).

Furthermore, we examined whether the molecular correlations observed in PDAC clinical specimens were also present in the KP-4 cell line, using knockdown cells for validation. In qRT-PCR analysis, MCM4 knockdown cell lines showed a significant reduction in MCM4 expression compared to cells transfected with control siRNA. Conversely, the expression levels of CLDN18 and PDX1 were significantly upregulated. In addition, KIFC1 expression was significantly suppressed, similar to the changes observed in MCM4 cells (Fig. 3E). These results revealed that at the cellular level, the knockdown of MCM4 suppressed pancreatic cancer progression and was associated with cancer stemness. Additionally, MCM4 was positively correlated with KIFC1 and negatively correlated with CLDN18 and PDX1.

### Single-cell analysis and MCM4 via in silico analysis

**Fig. 4:**
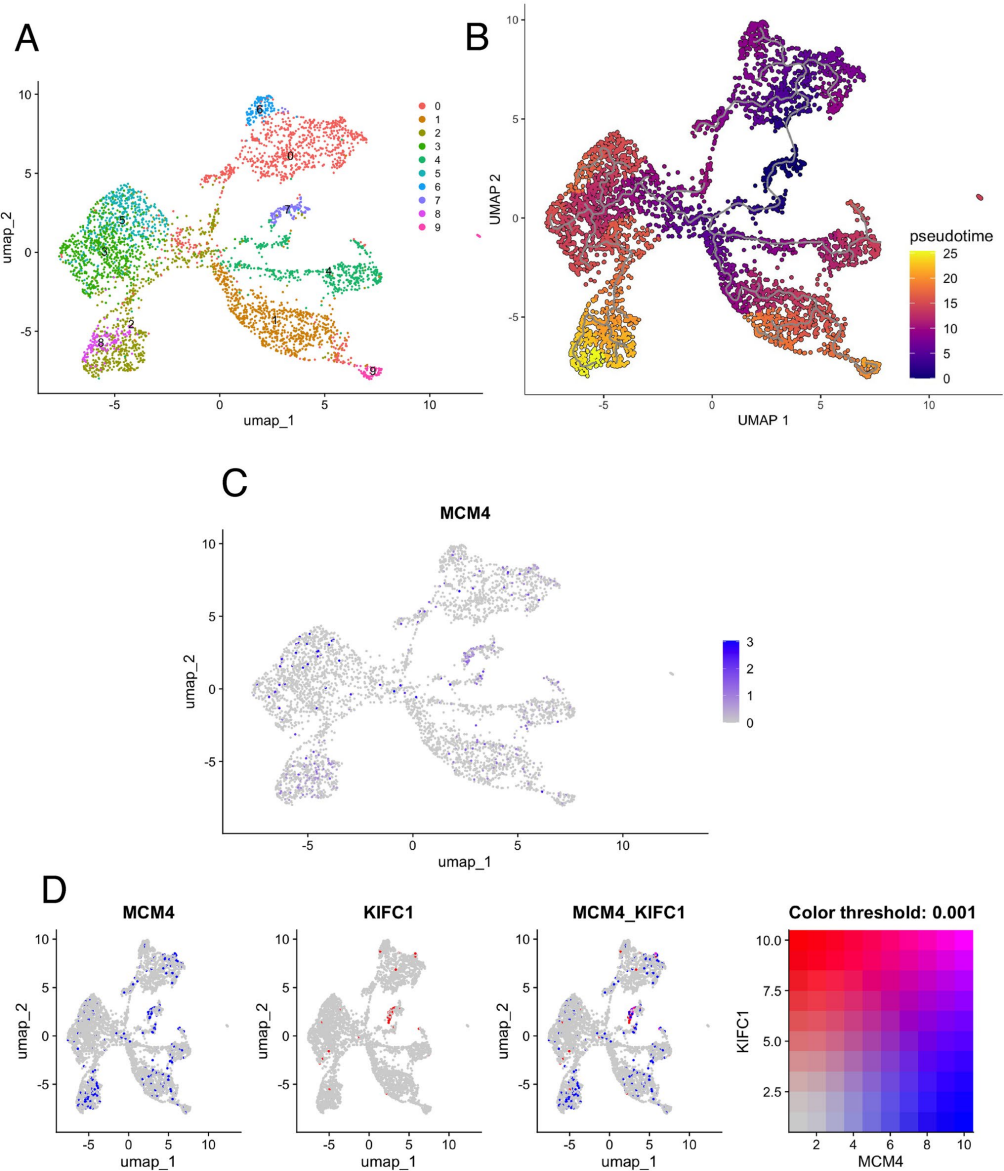
Single-cell analysis of six pancreatic ductal carcinoma cases based on GSE212966. **A** UMAP dimensional reduction analysis. **B** Trajectory analysis by Monocle3. **C** FeaturePlot displaying MCM4 expression across the identified cell populations in the UMAP. **D** FeaturePlot analysis of MCM4 and KIFC1 expression

**Fig. 5:**
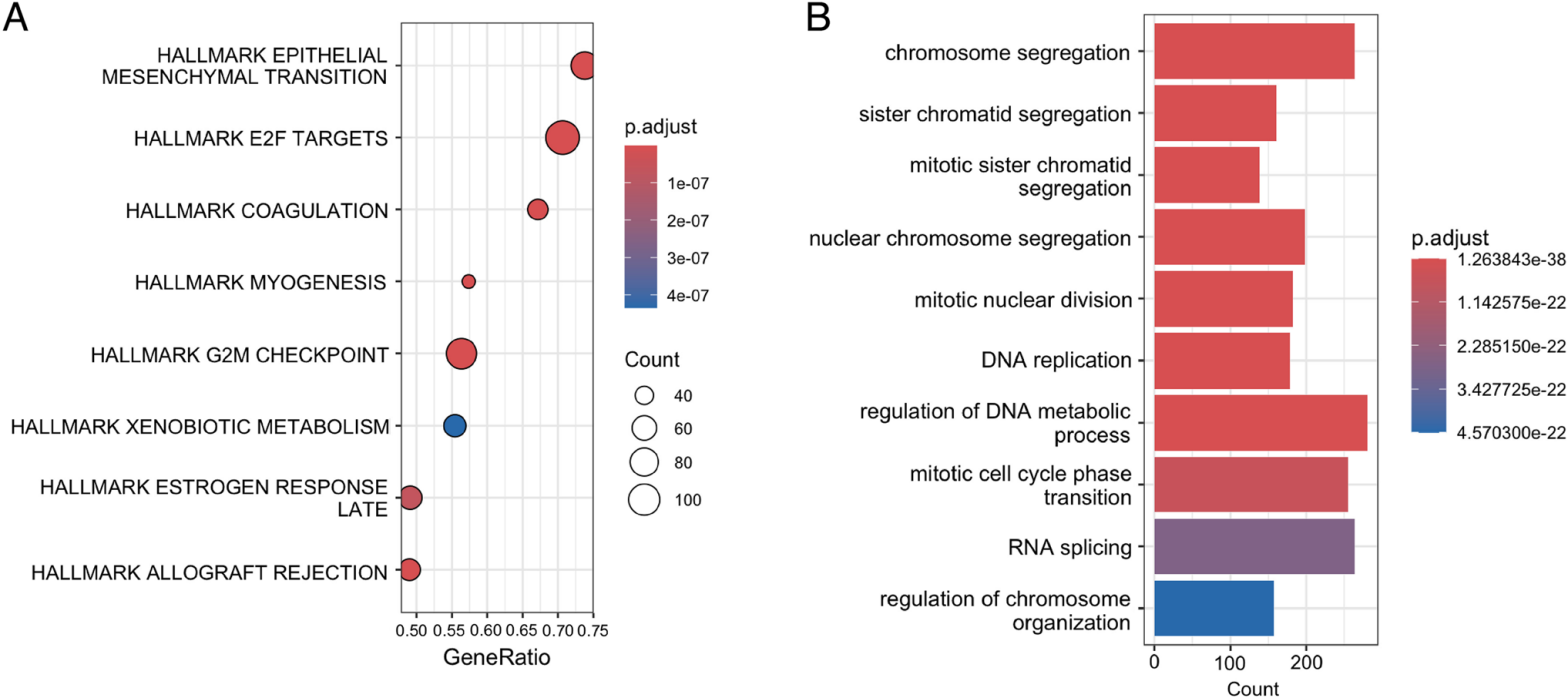
Pathway analysis of the focused cluster. **A** Gene set enrichment analysis of the MCM4 and KIFC1 co-expression cluster. **B** Gene ontology analysis of the MCM4 and KIFC1 co-expression cluster

Next, we analyzed single-cell RNA sequencing data from six PDAC clinical specimens available in a public database (GSE212966). From 26,735 cells, we extracted 4,260 cancer cells and performed dimensionality reduction using UMAP (Fig. 4A). When classified into clusters, cancer cells were divided into 10 clusters, ranging from clusters 0 to 9. Subsequently, we conducted a pseudo-time analysis using Monocle 3 (Fig. 4B) and compared the results with MCM4 expression (Fig. 4C). Cluster 7 was positioned upstream of the pseudo-time trajectory, where MCM4 expression was notably enriched. Moreover, MCM4 and KIFC1 were coexpressed in some cells within Cluster 7 (Fig. 4D). GSEA based on the gene expression of this cluster identified gene sets related to epithelial-mesenchymal transition, E2 F targets, and the G2M checkpoint (Fig. 5A). Gene ontology analysis revealed that genes involved in chromosome segregation and mitosis were prominently enriched (Fig. 5B). These results suggested that MCM4 may cooperate with KIFC1 in some cells within Cluster 7 to regulate the cell cycle, potentially influencing cancer stemness.

## Discussion/conclusion

This study demonstrated that MCM4 overexpression serves as an independent poor prognostic factor in clinical specimens of PDAC, showing a correlation with KIFC1 and Ki-67, further validating these findings at the cellular level. To our knowledge, this is the first report of immunohistochemical analysis of MCM4 in a large cohort. Our findings suggest a novel prognostic marker for PDAC using a modality routinely employed in pathological diagnostic practice.

In this study, immunohistochemical staining of MCM4 was performed using clinical specimens, revealing the nuclear localization of the staining and its correlation with pN grade. The nuclear localization of MCM4 was consistent with its role in DNA replication [14]. Additionally, MCM4 expression was found to be associated with poor prognosis and was identified as an independent prognostic factor. In this study, a 5% cutoff was also used, consistent with our previous investigation [21]. These findings suggest that even a small population expressing MCM4 may contribute to the progression of PDAC. Notably, similar results have been reported for multiple cancers [28, 29], including those in our previous studies [20, 21]. Here, poor prognosis associated with MCM4 expression in clinical specimens was attributed to metastasis, which involves multiple steps [30], such as epithelial–mesenchymal transition (EMT) and invasion. Our analysis using cancer cell lines revealed changes in the proliferative capacity and spheroid formation ability. Therefore, in a larger number of cases, a significant correlation with tumor size may also be evident.

This study raises important questions about the molecules and functions of MCM4. Notably, MCM4 expression correlated with KIFC1 expression while being inversely associated with the gastric phenotype markers CLDN18 and PDX1. These findings were confirmed not only in clinical specimens but also by qRT-PCR analysis using knockdown PDAC cell lines, which produced similar results. Furthermore, single-cell analysis revealed the co-expression of KIFC1 and MCM4, supporting the robustness of this relationship. The gastric phenotype is commonly used in the classification of gastric cancer [31]. In pancreatic cancer, the expression of the representative gastric marker MUC5 AC is known to be associated with poor prognosis [32]. Additionally, our previous studies reported that ANXA10 is linked to the gastric phenotype [33] and that its upregulation in pancreatic cancer is associated with poor prognosis [25]. MCM4 may contribute to pancreatic cancer malignancy through a mechanism distinct from that of gastric phenotype markers. However, the relationship between MCM4 and markers of the gastric-type phenotype has not been investigated for all relevant markers. Therefore, further detailed studies will be necessary in the future.

Notably, a correlation between KIFC1 and MCM4 has not been previously reported, making this study the first to highlight this relationship. Pathway analysis of the cell clusters coexpressing these genes revealed alterations in gene sets related to EMT as well as genes involved in DNA replication. *KIF2 C* and *MCM4* have been identified as hub genes [34], suggesting a similar relationship between *KIFC1* and *MCM4*. MCM4 may be involved in lymph-node metastasis based on clinical specimen analysis, potentially through its role in altering the surrounding microenvironment or influencing EMT. However, these observations remain speculative, and further investigation using in vitro or in vivo models is necessary to confirm the relationship between MCM4 and lymph-node metastasis.

This study had several limitations. First, it was a retrospective study. Prospective trials are necessary if treatments targeting MCM4 are to be pursued. Second, only patients from a single institution were included. Given that the prognosis of PDAC in this cohort was relatively favorable, there is a possibility of bias. In particular, when evaluating the proportional hazards’ model, multicollinearity was observed, resulting in the retention of pN stage and MCM4 expression as prognostic factors. With a larger sample size, comparisons with pStage and the potential inclusion of resection margin as a prognostic factor may become feasible. However, these limitations were mitigated by the use of experimental data from PDAC cell lines and single-cell analyses, ensuring that the novelty of these findings is unquestionable.

In summary, the present study demonstrated the significance of MCM4 expression in PDAC and its association with cancer stemness. Furthermore, our findings suggested that MCM4 immunohistochemical staining could serve as a potential novel prognostic and predictive biomarker for patients with pancreatic cancer.

## Data Availability

Data used to support the findings of this study are available from the corresponding author upon request.
